# Bioinformatics analysis of potential molecular markers and immunological characteristics shared between post-treatment Lyme disease syndrome and rheumatoid arthritis

**DOI:** 10.3389/fimmu.2026.1796178

**Published:** 2026-07-07

**Authors:** Yantong Chen, Xin Guo, Xinyuan Xu, Meng Liu, Yanshuang Luo, Shunli Cai, Huangjuan Zhao, Yan Dong, Guozhong Zhou

**Affiliations:** 1 Faculty of Life Science and Technology & The Affiliated Anning First People’s Hospital, Kunming University of Science and Technology, Kunming, Yunnan, China; 2 Department of Pain Medicine, The Affiliated Anning First People’s Hospital of Kunming University of Science and Technology, Kunming, Yunnan, China; 3 School of Basic Medical Sciences, Kunming University of Science and Technology, Kunming, Yunnan, China

**Keywords:** bioinformatics, immune imbalance, machine learning algorithms, post-treatment Lyme disease syndrome, rheumatoid arthritis

## Abstract

**Background:**

Post-treatment Lyme disease syndrome (PTLDS) and rheumatoid arthritis (RA) are both characterized by chronic inflammation and immune dysregulation; however, whether they share underlying molecular immune mechanisms remains unclear.

**Methods:**

Peripheral blood mononuclear cell (PBMC) transcriptomic datasets from the GEO database were analyzed to identify common differentially expressed genes (DEGs) between PTLDS and RA. Gene Ontology (GO) and Kyoto Encyclopedia of Genes and Genomes (KEGG) enrichment analyses were subsequently performed. Feature genes were screened using least absolute shrinkage and selection operator (LASSO) regression and support vector machine-recursive feature elimination (SVM-RFE) machine learning algorithms. Gene set enrichment analysis (GSEA) and xCell analysis were conducted to characterize signaling pathway features and immune cell infiltration patterns in both diseases. In addition, prediction-based ceRNA regulatory network analysis and drug association analysis were performed as exploratory components.

**Results:**

A total of 44 common DEGs were identified, which were primarily enriched in neutrophil chemotaxis, complement activation, and immune-inflammatory processes. Machine learning analysis ultimately identified ZNF83 as the feature gene. GSEA revealed that both diseases were associated with innate immune-related pathways and immunoregulatory processes and exhibited certain similarities in immune cell composition. ZNF83 showed preliminary discrimination between disease and control samples in both PTLDS and RA (AUC = 0.934 and 0.765, respectively), although these estimates require validation in larger independent cohorts. Furthermore, DSigDB analysis suggested potential associations between ZNF83 and several candidate therapeutic compounds.

**Conclusion:**

PTLDS and RA share certain common features at the level of immune-inflammatory regulation, and ZNF83 may serve as a potential feature regulatory molecule involved in the immune processes of both diseases. This study provides new insights into the immunopathological mechanisms of PTLDS and its relationship with autoimmune diseases.

## 1 Introduction

Lyme disease (LD) is a zoonotic disease caused by infection with *Borrelia burgdorferi* (Bb) (1). Since it was first reported in the 1970s (2), Lyme disease has been identified in more than 70 countries worldwide and is predominantly prevalent in temperate regions of North America, Europe, and Asia (3). It is estimated that more than 476, 000 new cases occur annually in the United States (4), while approximately 129, 000 cases are reported each year in Europe (5). The clinical manifestations of Lyme disease are diverse and may involve multiple organ systems, including the skin, nervous system, heart, and joints (1). Although most patients recover completely following antibiotic treatment, a subset continue to experience persistent symptoms such as fatigue, pain, and cognitive impairment after the infection has resolved. When these symptoms persist for more than six months, the condition is referred to as post-treatment Lyme disease syndrome (PTLDS) (6).

Currently, the pathogenesis of PTLDS remains incompletely understood. However, multiple studies have suggested that it may be associated with factors such as persistent immune stimulation induced by Bb or residual antigens following infection, impaired immune regulation, and abnormal autoimmune responses (6–8). Existing research indicates that PTLDS patients exhibit significant immune abnormalities, including elevated levels of anti-neurological antibodies, persistent activation of chronic inflammatory signaling, and underlying autoimmune responses (9, 10). This suggests that the persistent symptoms may be closely related to post-infection immune response imbalance and abnormal activation of autoimmune mechanisms. Specifically, Chandra et al. (9) found significantly higher anti-neuronal antibody levels in PTLDS patients compared to healthy controls and recovered Lyme disease patients. Additionally, other studies observed more specific immune dysregulation in PTLDS patients, including altered T-cell subset proportions, persistent chronic inflammatory signaling, and potential disruption of immune tolerance, further highlighting their immune imbalance (10). However, another study on Rheumatoid Factor (RF) and Anti-Citrullinated Protein Antibodies (ACPA) revealed that the positivity rates for these two classic autoimmune arthritis-associated antibodies in PTLDS patients were comparable to those in the general population, suggesting that their joint symptoms do not align with the serological characteristics of RA-type autoimmune arthritis (11). Comprehensive evidence indicates that while PTLDS involves persistent immune dysregulation, its immunophenotype exhibits both overlaps and significant differences from classic autoimmune arthritis.

Rheumatoid arthritis (RA) is a prototypical chronic inflammatory autoimmune disease characterized by symmetrical polyarthritis, synovial hyperplasia, and progressive joint destruction (12, 13). Beyond the joints, RA can involve organs such as the lungs, cardiovascular system, and eyes, triggering systemic inflammatory responses and multi-organ damage (14). Due to its well-defined immunopathological mechanisms, RA is considered a crucial model for studying post-infectious autoimmune abnormalities (12, 13). Therefore, comparing the immune characteristics of PTLDS with RA facilitates a more systematic characterization of its immune alterations and provides insights into potential immune regulatory mechanisms.

In recent years, an increasing number of studies have utilized bioinformatics and machine learning approaches to explore the molecular mechanisms and potential therapeutic targets of various diseases. For example, previous studies based on the analysis of cuproptosis-related and ferroptosis-related genes have revealed the immune-inflammatory characteristics and potential regulatory networks in RA (15, 16). These findings suggest that integrated bioinformatics analyses contribute to the systematic elucidation of disease-related immune regulatory mechanisms and provide new perspectives for investigating disease pathogenesis.

Based on this, the present study focused on PTLDS and RA and obtained high-throughput sequencing data of peripheral blood mononuclear cells (PBMCs) from patients with both diseases through the GEO database to perform a systematic bioinformatics analysis. Gene Ontology (GO) and Kyoto Encyclopedia of Genes and Genomes (KEGG) enrichment analyses were conducted for differentially expressed genes (DEGs). In addition, least absolute shrinkage and selection operator (LASSO) regression and support vector machine-recursive feature elimination (SVM-RFE) machine learning algorithms were employed to identify feature genes. Gene set enrichment analysis (GSEA) was subsequently performed for the identified feature gene. Furthermore, immune cell infiltration characteristics were evaluated for both diseases, while prediction-based ceRNA regulatory network analysis and drug association analysis were used as exploratory analyses.

The present study aims to systematically compare PTLDS and RA at both the molecular and immunological levels, in order to clarify whether PTLDS exhibits biological characteristics of autoimmune diseases and to explore potential shared regulatory mechanisms between the two conditions, thereby providing a theoretical basis for a deeper understanding of the immunopathological processes of PTLDS and its potential therapeutic targets.

## 2 Materials and methods

### 2.1 Data source

RNA-seq datasets for PTLDS and RA were retrieved from the Gene Expression Omnibus (GEO) database. For PTLDS analysis, 7 PTLDS case samples were extracted from GSE77929. Because GSE77929 did not provide an independent healthy control group, 13 healthy control samples were obtained from GSE63085. GSE63085 was generated by the same research team, used the same GPL11154 platform and PBMC RNA-seq experimental system, and included 29 patients with Lyme disease and 13 matched healthy controls. In that original study, persistent symptoms and PTLDS status were evaluated at visit 5 (V5), 6 months after treatment completion. Therefore, the GSE63085 healthy controls were considered a reasonable control source for the GSE77929 PTLDS V5 samples. Data normalization was performed prior to downstream analysis to minimize the impact of technical heterogeneity. For RA analysis, 20 untreated RA case samples and 10 matched healthy control samples were extracted from GSE229449. The correspondence between dataset accession numbers and disease groups was checked throughout the Methods, figure legends, and data availability statement.

### 2.2 Differentially expressed gene screening

The R software (v4.3.2) was used to perform data normalization of PTLDS data using the normalizeBetweenArrays function. Subsequently, the Limma package was used to perform differential expression analysis on the PTLDS and RA datasets, respectively (17). DEGs were identified using a threshold of P value < 0.05 and |logFC| ≥ 0.5. Volcano plots and heatmaps were generated using base plotting functions in R and the pheatmap package to visualize the expression patterns of DEGs. Finally, a Venn diagram of DEGs between the two diseases was constructed using the jvenn online analysis tool to identify common DEGs (18).

### 2.3 GO and KEGG enrichment analysis

Enrichr is a comprehensive gene set enrichment analysis platform. To explore the potential biological functions and signaling pathways of the common DEGs in the two diseases, the present study used the Enrichr online tool to perform GO functional and KEGG pathway enrichment analyses of the common DEGs (19). The top 10 biological process (BP), cellular component (CC), and molecular function (MF) terms with P value < 0.05 in the GO analysis were extracted separately for visualization. In addition, significantly enriched pathways from the KEGG, WikiPathways, and Reactome Pathways databases were analyzed and visualized (19, 20).

### 2.4 Screening feature gene via machine learning algorithms

To further identify the hub gene, LASSO regression and SVM-RFE were applied to perform feature selection on the common DEGs, and the intersection of the results from the two algorithms was defined as the final hub gene (21). Subsequently, receiver operating characteristic (ROC) curves were used to preliminarily evaluate the ability of the hub gene to distinguish between disease and control groups, and the area under the curve (AUC) was used as the performance metric (22, 23).

### 2.5 Gene set enrichment analysis

To further explore the potential biological functions and signaling pathways associated with feature gene, the present study performed gene set enrichment analysis using the GSEA function in the clusterProfiler package in R software. The gene sets used were c2.cp.kegg.symbols.gmt and c5.go.symbols.gmt (24, 25). All genes were ranked according to their logFC values, and a threshold of P value < 0.05 was used as the criterion for significant enrichment to identify significantly enriched functions and pathways. Finally, the top six enriched results were visualized.

### 2.6 Analysis of immune cell infiltration in feature gene

To evaluate immune cell infiltration characteristics, the present study analyzed transcriptome expression matrices using the xCell package in R software (26). xCell is based on gene signature sets for 64 immune and stromal cell types and applies an extended algorithm derived from single-sample gene set enrichment analysis (ssGSEA) to quantify the relative abundance of each cell type. In addition, it incorporates a spillover compensation method to correct for signal interference between different cell types, thereby generating comparable cell abundance scores (27).

### 2.7 Drug prediction

To identify potential drug intervention clues associated with feature gene, the present study performed drug enrichment analysis of feature gene based on the DSigDB database within the Enrichr platform to screen candidate drugs that may have regulatory effects (28).

### 2.8 Prediction of ZNF83-related ceRNA regulatory networks

Because ceRNA analysis is a prediction-based and hypothesis-generating approach, it was used only to explore potential upstream regulatory clues for ZNF83. miRNA-mRNA interactions were first predicted based on the TargetScan, miRanda, and miRDB databases to identify miRNAs that may potentially bind to ZNF83 (29). Subsequently, spongeScan was used to predict lncRNA-miRNA interactions, and potential ceRNA regulatory axes were constructed. The resulting network was visualized and analyzed using Cytoscape (v3.10.0) (30). These predicted interactions were not interpreted as evidence of direct causal regulation.

### 2.9 Real-time quantitative PCR analysis of cell models

The human monocyte leukemia cell line THP-1 was cultured in RPMI 1640 medium supplemented with 10% fetal bovine serum and 1% penicillin-streptomycin at 37 °C under 5% CO_2_ conditions. THP-1 cells were seeded into 6-well plates and induced with 100 nM Phorbol 12-myristate 13-acetate (PMA) for 24 hours. Subsequently, cells were stimulated with Bb (MOI = 1) or LPS (100 ng/mL) for 24 hours as two separate stimulation conditions rather than a combined treatment. Bb stimulation was used to model infection-related innate immune activation relevant to PTLDS, whereas LPS stimulation was used as a simplified inflammatory macrophage activation model relevant to RA-associated innate immune pathways. This model was used to evaluate whether ZNF83 expression responds to inflammatory innate immune activation, not to reproduce the complete RA synovial microenvironment.

RNA was extracted from cell samples using Trizol reagent according to the manufacturer’s protocol. cDNA was synthesized using Yeasen Hifair^®^ III reverse transcriptase (Catalog No: 11141ES60), followed by quantitative real-time PCR with Hieff UNICON^®^ Universal Blue qPCR SYBR Master Mix (Catalog No: 11184ES08). GAPDH (Sangon, Catalog No: B661104-0001) was used as the internal reference gene, and results were analyzed using the 2^(-ΔΔCt) method. P < 0.05 was considered statistically significant. PCR primers were provided by Beijing Qingke Biotechnology (**Table 1**).

**Table 1:** Primer sequence.

Gene	Forward (5’-3’)	Reverse (3’-5’)
*ZNF83*	CCCGCAGTCACATCTACCAG	ATACGTTGGGGTGGGGAAAC
*GAPDH*	TGT TGC CAT CAA TGA CCC CT	TCG CCC CAC TTG ATT TTG GA

### 2.10 Statistical analysis

All statistical analyses and data visualization were performed using R software (v4.3.2), Cytoscape (v3.10.0), and GraphPad Prism 8.3. Independent samples t-test or Wilcoxon rank-sum test was used for comparisons between continuous variables. P < 0.05 was considered statistically significant.

## 3 Results

### 3.1 Screening of commonly differentiated genes between PTLDS and RA

**Figure 1:**
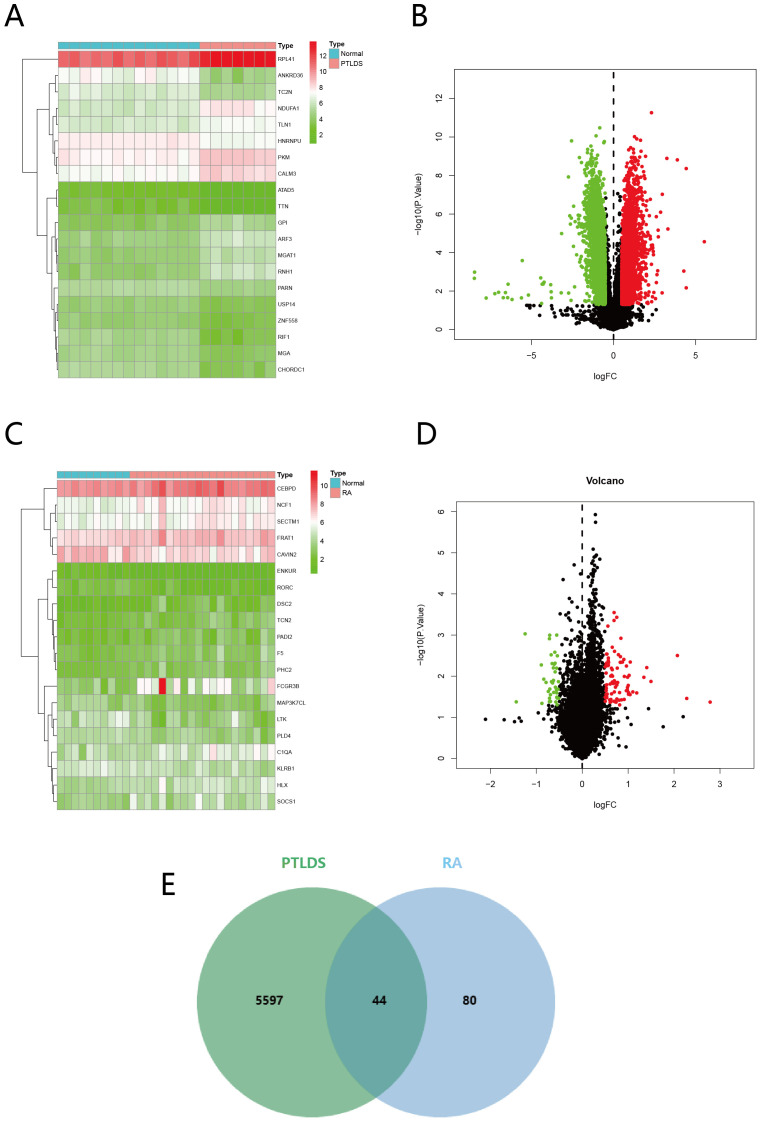
Differentially expressed gene analysis results. **(A, B)** Heatmap and volcano plot of differentially expressed genes in PTLDS samples. **(C, D)** Heatmap and volcano plot of differentially expressed genes in RA samples. **(E)** Venn diagram of the intersection of differentially expressed genes between PTLDS and RA. In the volcano plots, red indicates upregulated genes and green indicates downregulated genes.

To explore the potential molecular associations between PTLDS and RA, differential expression analysis was performed on transcriptome data obtained from the GEO database. The limma package was used to identify DEGs in the two datasets, and corresponding volcano plots and heatmaps were generated (**Figures 1A–D**). In the PTLDS dataset, a total of 5, 641 DEGs were identified, including 2, 807 upregulated and 2, 834 downregulated genes (**Figure 1B**). In the RA dataset, 124 DEGs were identified, including 85 upregulated and 39 downregulated genes (**Figure 1D**). Furthermore, an intersection analysis of the two DEG sets was performed using the online tool Jvenn, resulting in 44 common DEGs (**Figure 1E**).

### 3.2 GO and KEGG functional and pathway enrichment analysis

**Figure 2:**
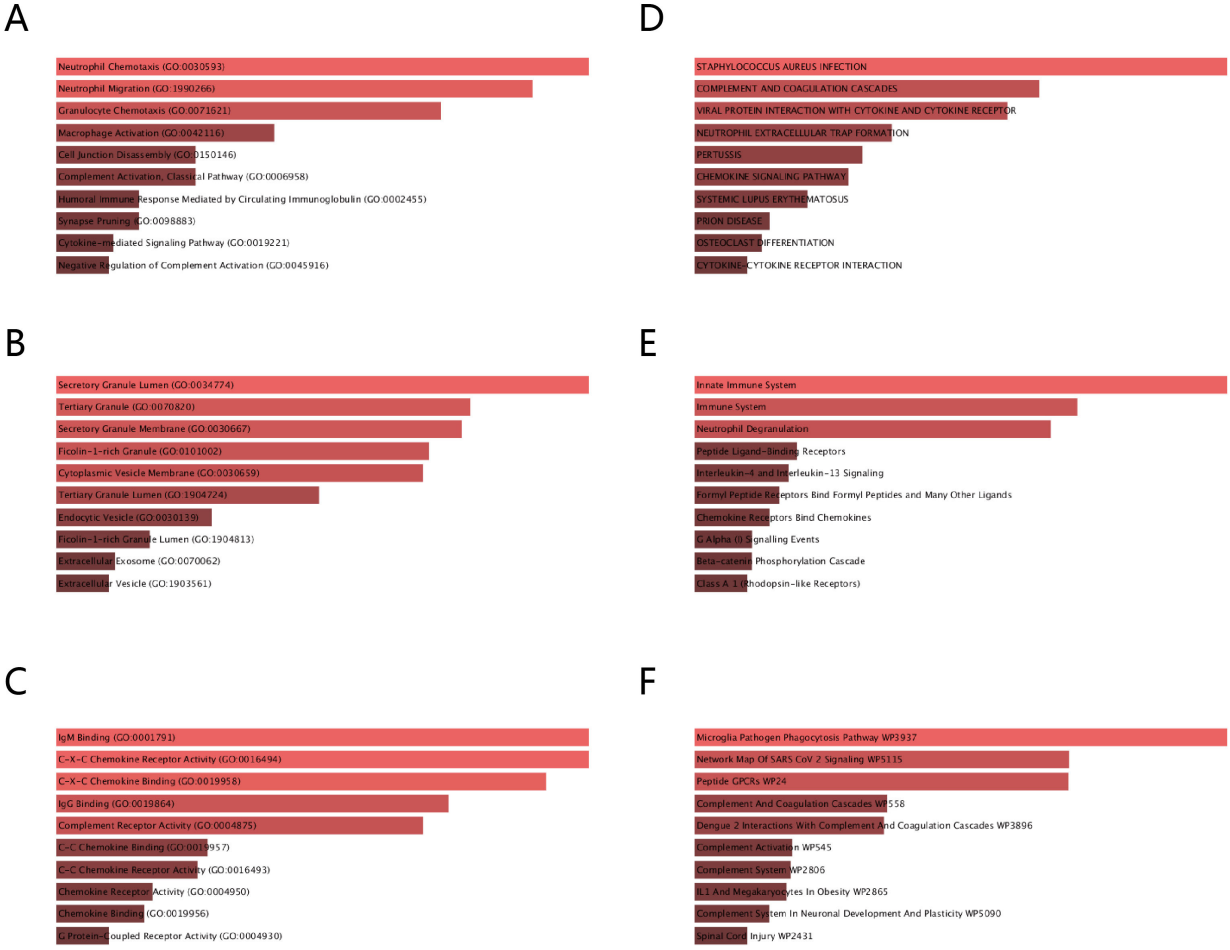
Functional and pathway enrichment analysis of common differentially expressed genes in PTLDS and RA. **(A–C)** GO enrichment analysis of common DEGs, including biological process (BP), cellular component (CC), and molecular function (MF). **(D)** KEGG pathway enrichment analysis. **(E)** Reactome pathway enrichment analysis. **(F)** WikiPathways pathway enrichment analysis.

The Enrichr online analysis tool was used to perform GO and KEGG enrichment analyses of the common DEGs between PTLDS and RA to clarify their potential biological functions and signaling pathway characteristics. GO enrichment analysis showed that, at the biological process (BP) level, the common DEGs were mainly enriched in immune-related processes, including neutrophil chemotaxis and migration, granulocyte recruitment, macrophage activation, complement activation, and cytokine-mediated signaling transduction, as well as humoral immune response and antigen-dependent immune regulation (**Figure 2A**). At the cellular component (CC) level, these genes were mainly enriched in secretory granule lumen, endocytic vesicles, and extracellular vesicles, suggesting their potential involvement in inflammatory mediator release and intercellular immune communication (**Figure 2B**). At the molecular function (MF) level, they were mainly associated with chemokine binding and receptor activity, complement receptor activity, and immunoglobulin binding, indicating important roles in immune cell recruitment and inflammatory response regulation (**Figure 2C**).

Pathway enrichment analysis showed that, in the KEGG database, the common DEGs were mainly enriched in the chemokine signaling pathway, neutrophil extracellular trap (NET) formation, complement and coagulation cascades, and cytokine–cytokine receptor interaction (**Figure 2D**). In the Reactome database, they were mainly enriched in the innate immune system, neutrophil degranulation, and G alpha (i)-related signaling pathways (**Figure 2E**). In WikiPathways, they were mainly involved in complement and coagulation cascades, the classical complement activation pathway, and peptide GPCR signaling pathways (**Figure 2F**).

### 3.3 Screening feature genes using multiple machine learning algorithms

**Figure 3:**
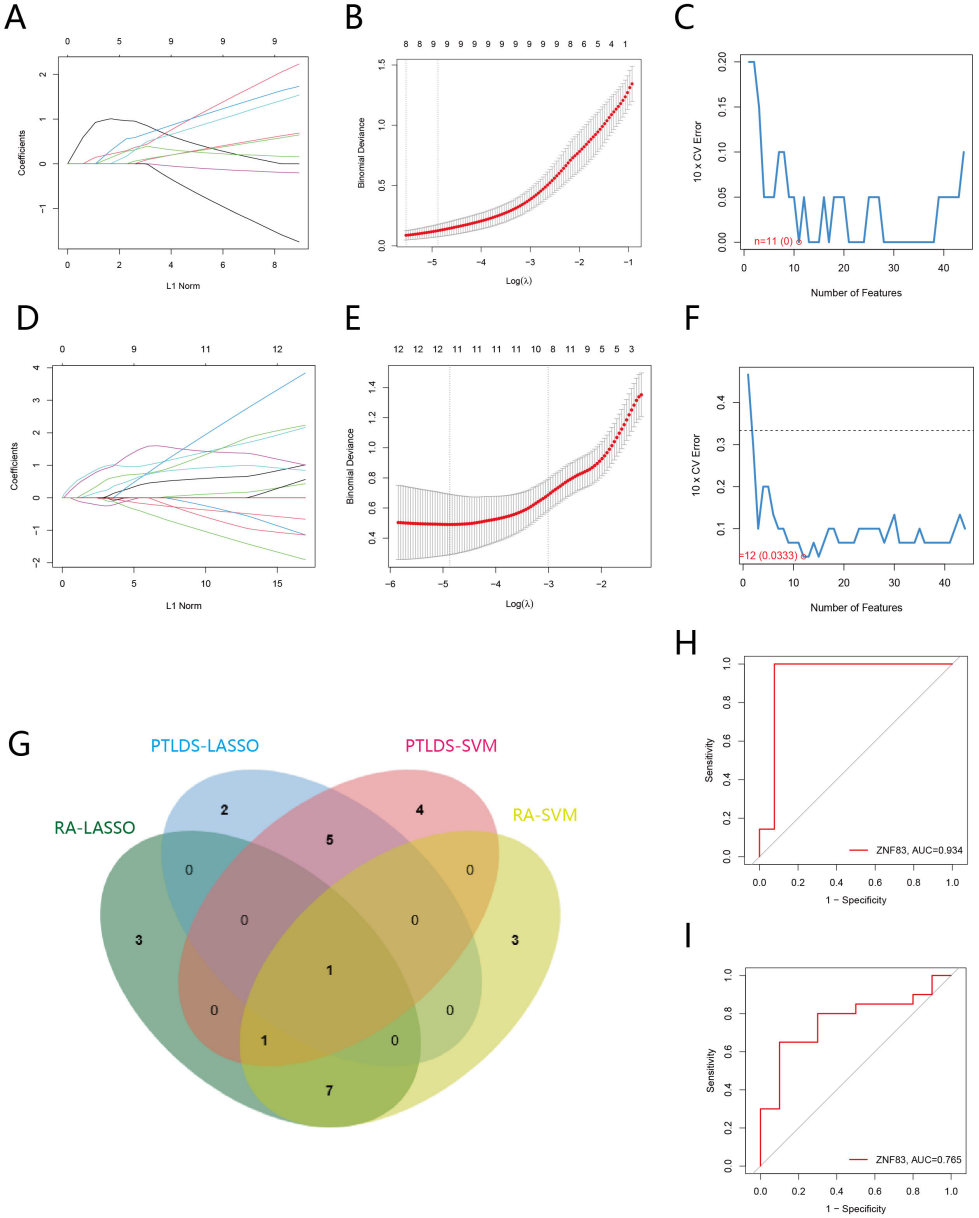
Identification of common feature genes in PTLDS and RA using LASSO regression and SVM-RFE algorithms. **(A, B)** LASSO regression analysis in PTLDS. **(C)** SVM-RFE analysis results in PTLDS. **(D, E)** LASSO regression analysis in RA. **(F)** SVM-RFE analysis results in RA. **(G)** Venn diagram of genes identified by LASSO regression and SVM-RFE in PTLDS and RA. **(H)** ROC curve of the common feature gene in PTLDS. **(I)** ROC curve of the common feature gene in RA.

LASSO and SVM-RFE algorithms were used to identify common feature gene in PTLDS and RA (**Figures 3A–F**). By taking the intersection of the results from the two algorithms, one common hub gene, ZNF83, was finally obtained (**Figure 3G**). To evaluate the discriminative ability of ZNF83 in PTLDS and RA, ROC curves were constructed for validation. The results showed that the AUC of ZNF83 was 0.934 in the PTLDS dataset and 0.765 in the RA dataset (**Figures 3H, I**), suggesting that ZNF83 has a certain diagnostic discriminative ability in both diseases, although its performance in RA is relatively weaker, and its stability still requires further validation in independent cohorts.

### 3.4 ZNF83 expression analysis and inflammatory-stimulus response

**Figure 4:**
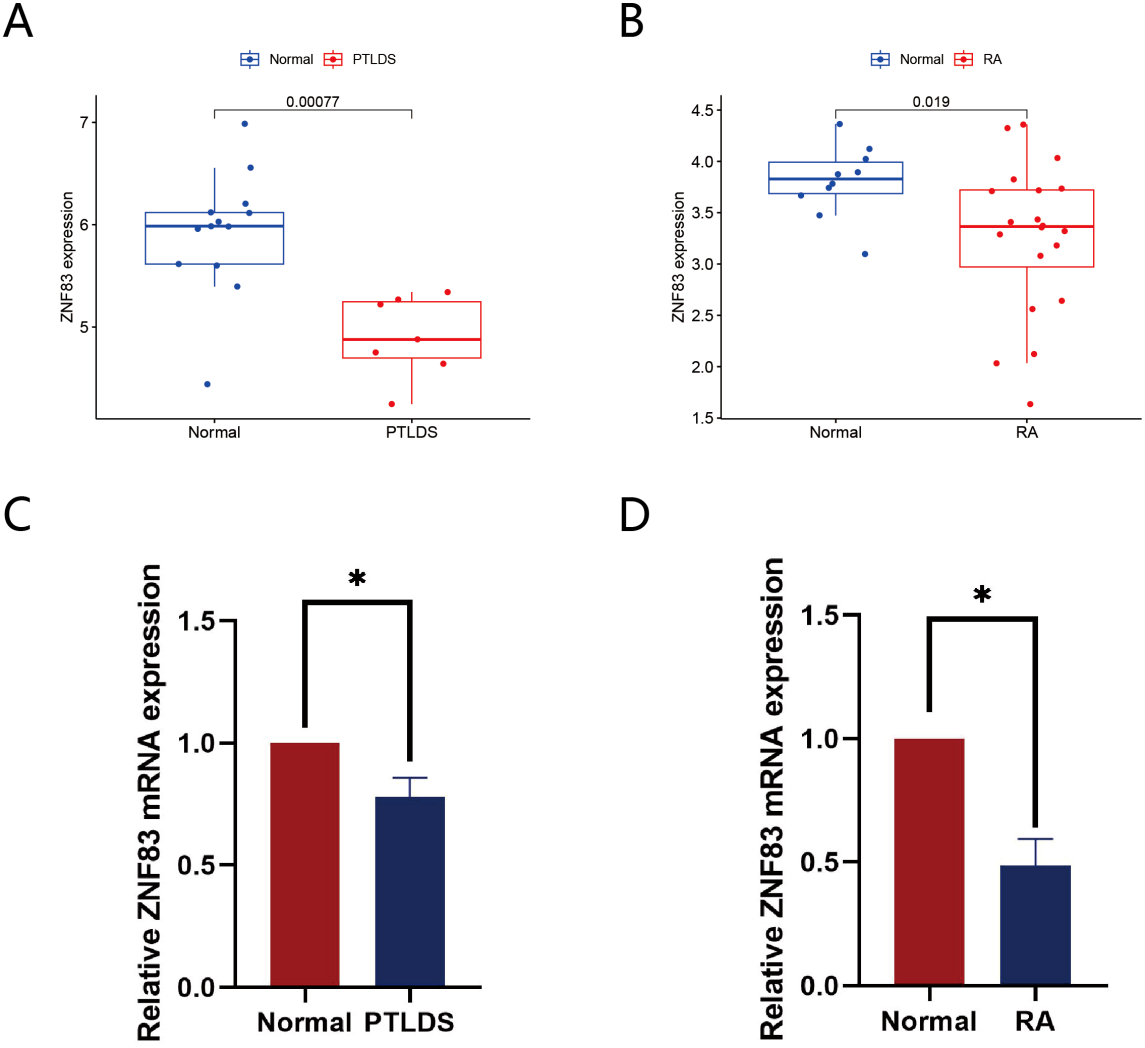
ZNF83 expression analysis and inflammatory-stimulus response. **(A)** Expression level of ZNF83 in the PTLDS dataset. **(B)** Expression level of ZNF83 in the RA dataset. **(C)** qRT-PCR analysis of ZNF83 expression under Bb stimulation in THP-1 cells. **(D)** qRT-PCR analysis of ZNF83 expression under LPS stimulation in THP-1 cells. *P < 0.05.

To further examine the expression characteristics of the candidate feature gene, the expression levels of ZNF83 were first compared between control and disease groups in both transcriptomic datasets (**Figures 4A, B**). ZNF83 was significantly downregulated in both disease groups compared with the corresponding control groups. In addition, THP-1 cells stimulated with Bb or LPS were used as preliminary inflammatory-stimulus models to assess whether ZNF83 expression changed under inflammatory conditions. ZNF83 expression was also decreased after inflammatory stimulation (**Figures 4C, D**). These cell-based results support an inflammation-associated expression response of ZNF83, but they do not recapitulate the full pathological processes of PTLDS or RA.

### 3.5 Gene set enrichment analysis

**Figure 5:**
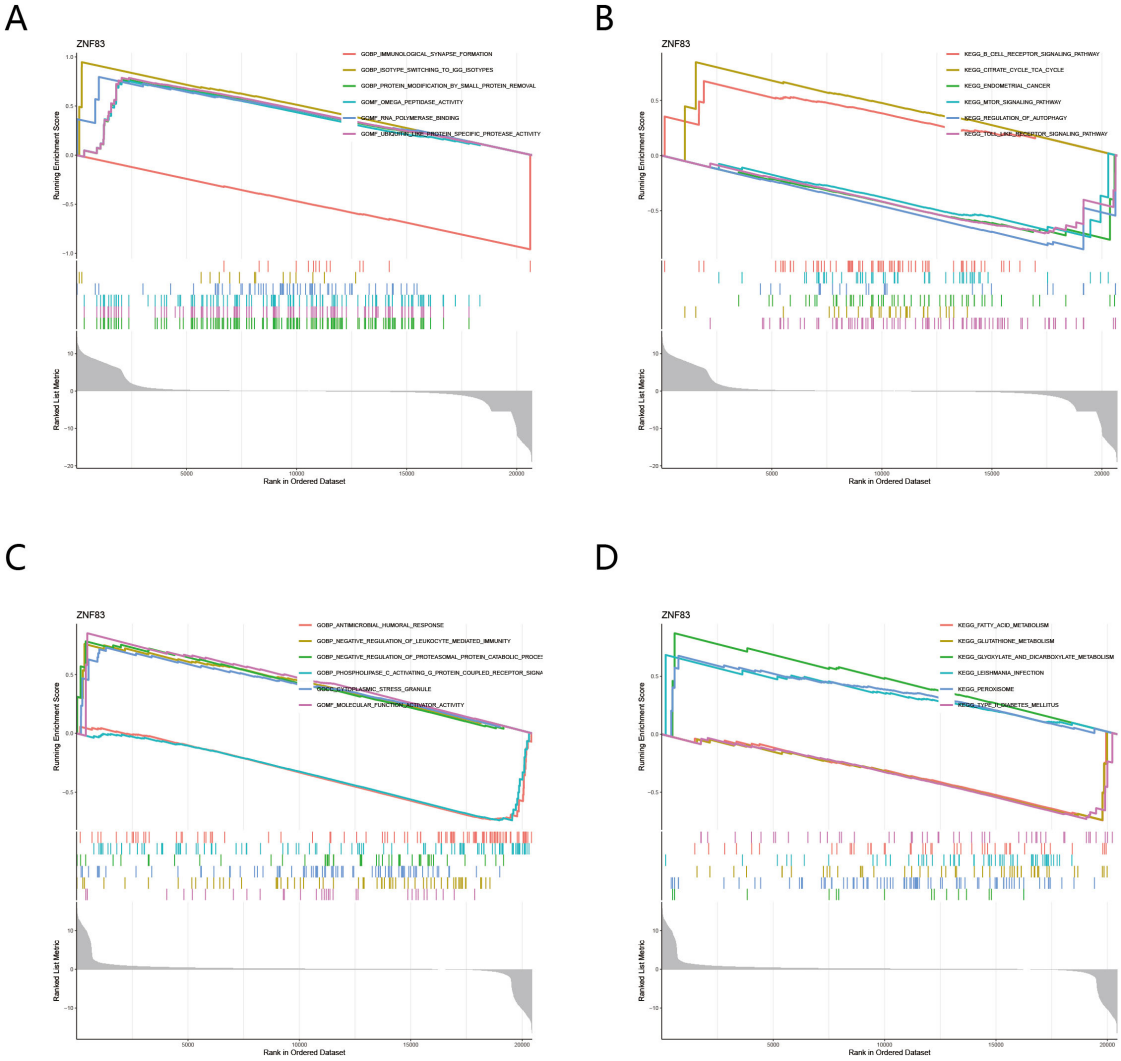
Gene set enrichment analysis (GSEA) of ZNF83-related gene sets. **(A)** GO-GSEA results of ZNF83 in PTLDS. **(B)** KEGG-GSEA results of ZNF83 in PTLDS. **(C)** GO-GSEA results of ZNF83 in RA. **(D)** KEGG-GSEA results of ZNF83 in RA.

GSEA results based on ZNF83 expression stratification showed distinct functional enrichment patterns between the high- and low-expression groups. In PTLDS, the ZNF83 high-expression group was mainly enriched in processes related to B cell receptor signaling pathway, IgG class switching, and tricarboxylic acid cycle, whereas the low-expression group was mainly enriched in inflammatory and immune-related pathways, including Toll-like receptor signaling pathway, mTOR signaling pathway, and autophagy regulation (**Figures 5A, B**). In RA, the ZNF83 high-expression group was mainly associated with proteasome regulation, stress granule formation, and peroxisome-related processes, whereas the low-expression group was mainly enriched in GPCR signaling pathway, fatty acid metabolism, and glutathione metabolism pathways (**Figures 5C, D**).

### 3.6 Immune cell infiltration

**Figure 6:**
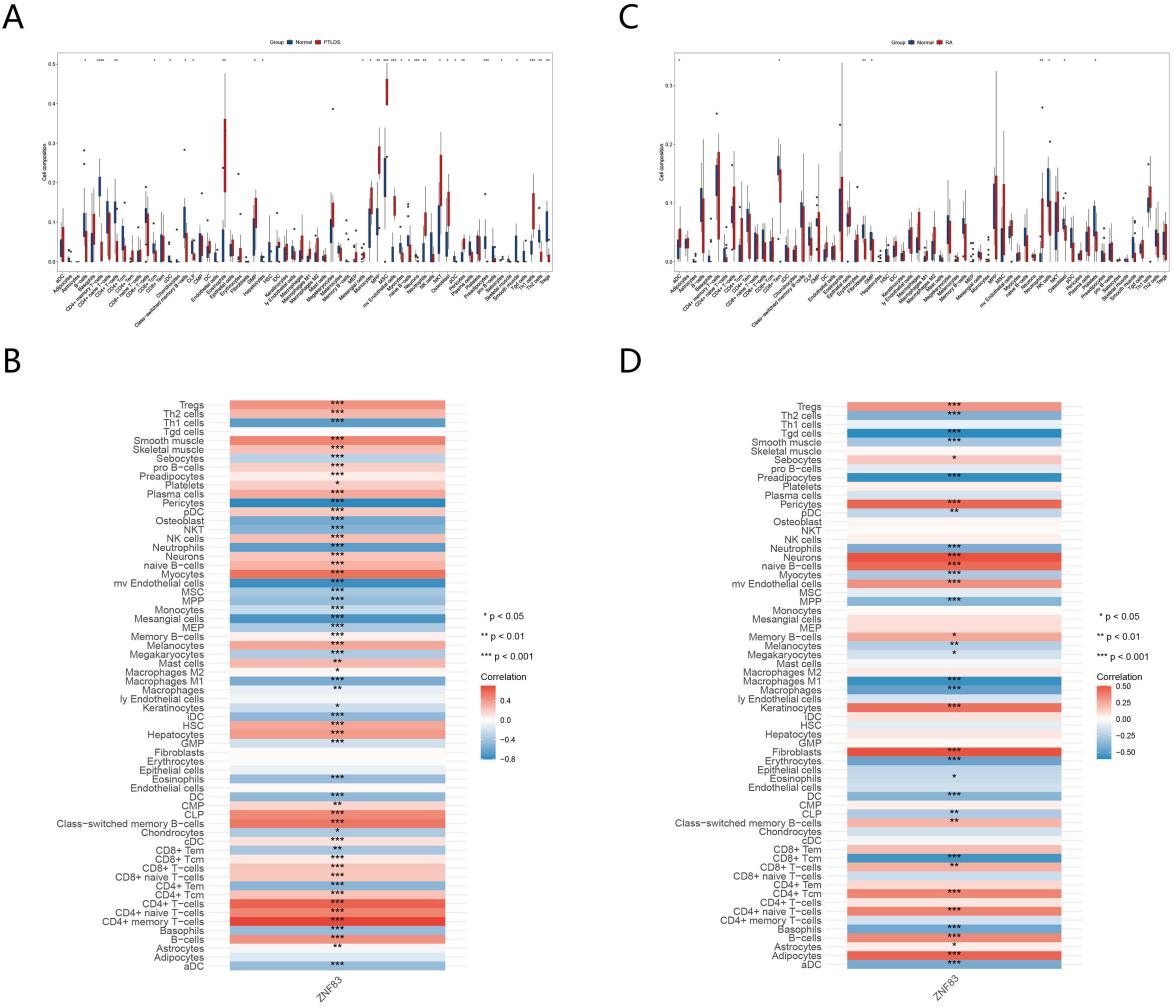
Immune cell infiltration analysis in PTLDS and RA. **(A)** Boxplot of immune cell infiltration levels in PTLDS samples. **(B)** Correlation heatmap between ZNF83 expression and immune cell infiltration levels in PTLDS. **(C)** Boxplot of immune cell infiltration levels in RA samples. **(D)** Correlation heatmap between ZNF83 expression and immune cell infiltration levels in RA.

In PTLDS, xCell immune infiltration analysis showed that, compared with the control group, neutrophils, GMP, MPP, MSC, NKT cells, and Th1 cells were significantly increased in the disease group, whereas B cells, class-switched memory B cells, naive B cells, CD4^+^ memory T cells, CD4^+^ T cells, CD8^+^ Tcm, Tregs, cDCs, and pDCs were significantly decreased, suggesting an immune infiltration pattern characterized by increased innate immune–related cells and decreased adaptive immune cells in PTLDS (**Figure 6A**). Correlation analysis showed that ZNF83 expression was significantly positively correlated with B cells, CD4^+^ T cells, CD4^+^ memory T cells, naive B cells, class-switched memory B cells, and Tregs, whereas it was significantly negatively correlated with neutrophils, M1 macrophages, Th1 cells, NKT cells, cDCs, and pDCs (**Figure 6B**).

In RA, aDCs and neutrophils were significantly increased in the disease group, whereas CD8^+^ Tem cells, GMP, NK cells, fibroblasts, osteoblasts, and platelets were significantly decreased, suggesting abnormal inflammatory cell infiltration and immune regulatory imbalance in RA (**Figure 6C**). Correlation analysis of ZNF83 showed that its expression was positively correlated with B cells, CD4^+^ naive T cells, CD8^+^ T cells, and Tregs, whereas it was negatively correlated with neutrophils, M1 macrophages, dendritic cells, and Tgd cells (**Figure 6D**).

### 3.7 Drug prediction

**Figure 7:**
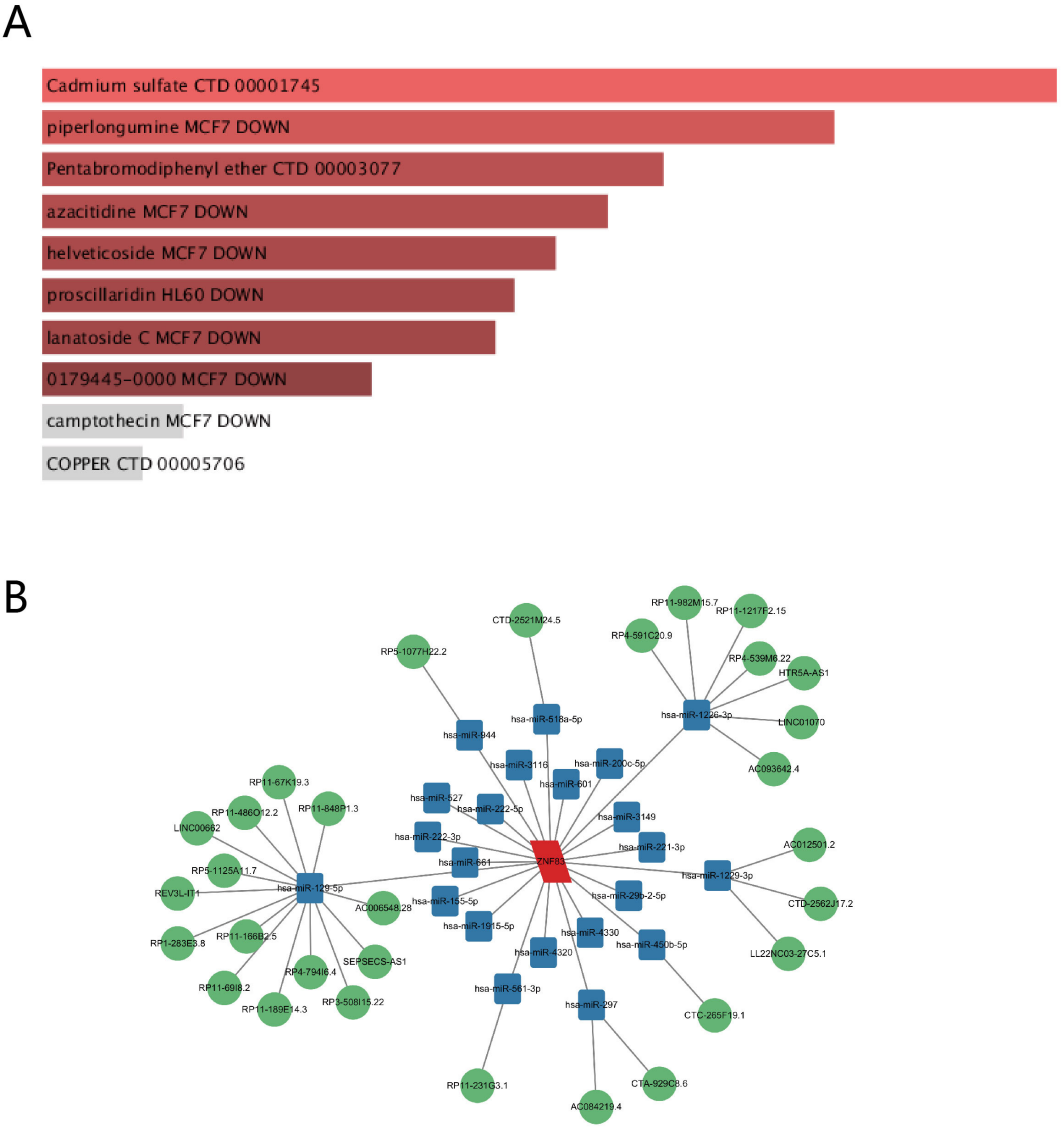
Drug prediction and prediction-based ceRNA network analysis of ZNF83. **(A)** Predicted candidate drugs associated with ZNF83. **(B)** Predicted ceRNA regulatory network based on ZNF83, illustrating potential regulatory relationships among ZNF83, miRNAs, and lncRNAs.

Drug enrichment analysis of ZNF83 was performed based on the DSigDB database within the Enrichr platform, and a total of eight candidate compounds significantly associated with ZNF83 were identified, including cadmium sulfate, piperlongumine, pentabromodiphenyl ether, azacitidine, helveticoside, proscillaridin, lanatoside C, and 0179445-0000 (**Figure 7A**). Among these, cadmium sulfate showed the most significant association with ZNF83 (P = 0.0072).

### 3.8 Prediction of the ZNF83-related ceRNA network

Prediction-based mRNA-miRNA and miRNA-lncRNA network analysis suggested that ZNF83 may have potential upstream regulatory links with several miRNAs, including hsa-miR-1226-3p, hsa-miR-129-5p, hsa-miR-155-5p, hsa-miR-200c-5p, and the hsa-miR-221/222 family (**Figure 7B**). These miRNAs were predicted to interact with multiple lncRNAs, forming a putative ceRNA regulatory network. Because these results are based on database prediction, they are presented as preliminary regulatory clues rather than direct evidence of biological regulation.

## 4 Discussion

This study aimed to explore the potential links between PTLDS and RA at the immunological and molecular levels. Previous studies have shown that patients with PTLDS exhibit persistent immune abnormalities and chronic inflammatory responses, whereas RA is a prototypical chronic autoimmune disease, and the two conditions share certain similarities in fatigue, joint pain, and abnormal inflammatory cytokine profiles (8, 12). However, whether the two diseases share common molecular regulatory mechanisms remains insufficiently investigated in a systematic manner. Therefore, based on PBMC transcriptome data from the GEO database, this study systematically investigated the shared molecular characteristics of PTLDS and RA using differential expression analysis, machine learning-based feature selection, functional enrichment analysis, immune infiltration analysis, GSEA, and ceRNA network construction, aiming to provide new insights into the potential mechanisms underlying persistent immune dysregulation in PTLDS.

Functional enrichment analysis of the common DEGs showed that PTLDS and RA shared genes were mainly enriched in inflammatory biological processes, including neutrophil chemotaxis and migration, macrophage activation, activation of the classical complement pathway, and cytokine-mediated signaling pathways. Meanwhile, KEGG, Reactome, and WikiPathways analyses indicated that the common DEGs were primarily involved in chemokine signaling pathways, neutrophil extracellular trap (NET) formation, complement and coagulation cascades, neutrophil degranulation, and the innate immune system. These findings suggest that both PTLDS and RA may be characterized by a persistent inflammatory state driven by myeloid cell activation and innate immune dysregulation. Previous studies have demonstrated that neutrophils play a critical role in chronic inflammation and tissue damage in RA, as they promote sustained autoimmune activation through the release of inflammatory mediators and NET formation (31). In addition, abnormal activation of the complement system has also been implicated in the amplification of chronic inflammation and immune-mediated tissue injury (32). Therefore, the present results suggest that persistent innate immune inflammatory responses may represent a shared immunological feature of PTLDS and RA.

To further identify shared feature genes between the two diseases, LASSO regression and SVM-RFE algorithms were applied, and ZNF83 was ultimately identified as the only common feature gene. ROC analysis suggested that ZNF83 had a preliminary ability to distinguish disease and control samples, although this result should be interpreted cautiously because of the limited sample size. ZNF83 was downregulated in both PTLDS and RA transcriptomic datasets. THP-1 cells stimulated with Bb or LPS were then used as inflammatory-stimulus models to examine whether ZNF83 expression was responsive to inflammatory conditions. Bb stimulation was used to model infection-related innate immune activation relevant to PTLDS, whereas LPS stimulation was used as a simplified inflammatory macrophage activation model relevant to RA-associated innate immune pathways. LPS-stimulated THP-1 cells or THP-1-derived macrophage-like cells have been used in RA-related studies to evaluate inflammatory macrophage activation, TLR4/NF-kappaB signaling, and cytokine production, and macrophage activation and M1/M2 imbalance are recognized contributors to RA synovial inflammation (33–35). The purpose of this experiment was not to fully recapitulate PTLDS or RA pathogenesis, but to provide preliminary support for the inflammation-associated expression pattern of ZNF83 suggested by the bioinformatics results.

GSEA analysis further revealed the potential biological functions associated with ZNF83. In PTLDS, the ZNF83 low-expression group was mainly enriched in inflammatory-related processes, including the Toll-like receptor (TLR) signaling pathway, mTOR signaling pathway, and autophagy regulation. Previous studies have shown that the TLR signaling pathway plays a feature role in pathogen recognition and innate immune activation, and its sustained activation can promote the production of inflammatory cytokines such as TNF-α and IL-6 through the MyD88/NF-κB axis (36). In addition, abnormalities in mTOR signaling and autophagy have been closely associated with persistent inflammation, metabolic imbalance, and disruption of immune homeostasis (37). In RA, ZNF83-associated enriched pathways were more related to lipid metabolism, glutathione metabolism, GPCR signaling, and humoral immune response. Previous studies have indicated that lipid metabolic reprogramming and redox imbalance can affect synovial cell activation and immune cell function, thereby contributing to the formation of the chronic inflammatory microenvironment in RA (38). The GPCR signaling pathway may also participate in immune regulation by modulating chemokine signaling and inflammatory mediator release (39).

The xCell immune infiltration analysis revealed marked alterations in immune cell composition in both PTLDS and RA, generally characterized by reduced adaptive immune cell populations and increased myeloid cell infiltration. Previous immune infiltration studies in RA have reported decreased levels of B cells, T cells, and NK cells, accompanied by increased levels of innate immune-related cells, including neutrophils, macrophages, and plasma cells (40, 41). Similarly, Girgis et al. reported reduced CXCR5^+^ naïve CD4^+^ T cells and increased CD8^+^ Th1-like cells in patients with PTLDS, suggesting persistent T-cell dysfunction and immune imbalance (42). These findings indicate that, despite their distinct etiologies, PTLDS and RA share common features of impaired adaptive immunity and enhanced myeloid cell activation.

Despite these shared characteristics, the patterns of immune dysregulation differed between the two diseases. In PTLDS, significant reductions in B cells, naïve B cells, and class-switched memory B cells were observed, accompanied by decreases in multiple CD4^+^ T-cell subsets, suggesting a tendency toward post-infectious immune dysregulation and systemic immune imbalance. Previous studies have likewise demonstrated persistent immune abnormalities and alterations in lymphocyte function in a subset of PTLDS patients even after pathogen clearance (42, 43). In contrast, alterations in fibroblasts, osteoblasts, and platelets were observed in RA. Aberrant fibroblast activation and disrupted bone metabolism are recognized as important pathological bases for joint destruction and tissue remodeling in RA (44, 45). These findings suggest that although PTLDS and RA share features of persistent inflammation and innate immune activation, their specific patterns of immune dysregulation are not identical. PTLDS appears to be more closely associated with post-infectious immune dysregulation, whereas RA is primarily characterized by tissue damage driven by classical autoimmune inflammation.

In the present study, increased neutrophil infiltration was observed in both diseases. Previous studies have shown that excessively activated neutrophils can promote inflammatory amplification and tissue damage through the release of inflammatory mediators, formation of neutrophil extracellular traps (NETs), and activation of the complement system (46). Consistently, our functional enrichment analyses demonstrated that both diseases were enriched in pathways related to neutrophil chemotaxis, neutrophil degranulation, and NET formation. Neutrophils are not only feature effector cells in inflammatory responses but can also contribute to the maintenance of the inflammatory microenvironment through interactions with monocytes/macrophages and lymphocytes (47). Therefore, although PTLDS and RA have distinct etiological backgrounds, the shared increase in neutrophil infiltration suggests that they may possess a common inflammation-amplifying mechanism driven by innate immunity, which may represent an important link between post-infectious chronic inflammation and autoimmune inflammation.

Given that ZNF83 was identified as the only shared feature gene in this study, we further investigated its potential role by integrating the GSEA and immune infiltration results. As a member of the C2H2-type zinc finger protein family, ZNF83 is considered to have DNA-binding and transcriptional regulatory potential; however, studies on ZNF83 remain limited, and existing reports have primarily focused on cancer-related contexts (48–50). ZNF83 has not been confirmed as a direct component or regulator of the TLR, autophagy, or immunometabolic pathways. Nevertheless, low ZNF83 expression was observed together with enrichment of inflammation- and metabolism-related pathways. Moreover, in both PTLDS and RA, ZNF83 expression was positively correlated with adaptive immune cells, including B cells, CD4+ T cells, and Tregs, whereas it was negatively correlated with neutrophils, M1 macrophages, and certain dendritic cell subsets. Combined with the GSEA and immune infiltration results, these findings raise the possibility that reduced ZNF83 expression may be associated with innate immune activation, inflammatory myeloid-cell enrichment, and altered immunometabolic status. This interpretation remains hypothesis-generating, and the underlying mechanisms require further validation through ZNF83 overexpression or knockdown experiments combined with assessments of TLR/NF-kappaB signaling activity, autophagic flux, and metabolism-related parameters.

Drug association analysis indicated that ZNF83 was potentially associated with multiple compounds, including piperlongumine, azacitidine, proscillaridin, and lanatoside C. Piperlongumine has been reported to possess biological activities related to the regulation of oxidative stress and inflammation-associated signaling pathways (51). As a DNA methylation inhibitor, azacitidine may exert its effects by inhibiting DNA methylation and modulating immune-related cellular responses (52). Proscillaridin and lanatoside C belong to the cardiac glycoside family, and previous studies have suggested that they may participate in the maintenance of ion homeostasis and influence oxidative stress- and inflammation-related signaling pathways (53). Combined with the GSEA results of this study, ZNF83-associated pathways involve processes such as TLR signaling, autophagy regulation, lipid metabolism, and oxidative stress. Therefore, the biological functions associated with these predicted compounds overlap to some extent with the pathways enriched for ZNF83, suggesting their potential immunomodulatory value. However, these associations are primarily based on database predictions, and the specific mechanisms involved require further experimental validation.

The ZNF83-related ceRNA analysis was retained as a limited, prediction-based component to provide potential upstream regulatory clues. Among the predicted miRNAs, hsa-miR-155-5p and hsa-miR-129-5p have been reported to be involved in inflammatory responses, immune cell activation, and autoimmune disease-related processes (54, 55). In particular, hsa-miR-155-5p can influence TLR/NF-kappaB signaling activity by regulating negative regulators such as SOCS1, thereby promoting inflammatory cytokine expression and sustained activation of innate immune responses (54). These observations suggest possible links between immune-related miRNA networks and ZNF83 expression, but the predicted ceRNA relationships remain hypothesis-generating and require experimental validation before mechanistic conclusions can be drawn.

This study has several limitations. First, the limited sample size, particularly in the PTLDS cohort, may affect the robustness and generalizability of the DEG lists, feature-gene screening, and ROC-based discrimination estimates. Therefore, the AUC results should be interpreted as preliminary evidence rather than definitive diagnostic or predictive performance. Second, this study was primarily based on analyses of public databases and lacks systematic *in vitro* and *in vivo* functional validation. The THP-1 stimulation experiments only evaluated the expression response of ZNF83 under inflammatory innate immune stimulation. Bb stimulation and LPS stimulation were used as simplified models of infection-related and inflammatory macrophage activation, respectively, and did not model the full pathological complexity of PTLDS or the RA synovial microenvironment. Finally, because the analyses were conducted using PBMC data, they may not fully reflect the immune status of local tissue microenvironments. Future studies integrating larger independent clinical cohorts, multi-omics datasets, and functional experiments are needed to further validate the specific role of ZNF83 in shared inflammatory pathways between PTLDS and RA and to explore its potential clinical value as a biomarker or therapeutic target.

## 5 Conclusion

This study systematically investigated the potential molecular links between PTLDS and RA using publicly available transcriptomic data and a series of bioinformatics approaches, including differential expression analysis, machine learning-based feature selection, functional enrichment analysis, immune infiltration analysis, GSEA, and ceRNA network construction. ZNF83 was identified as a shared feature gene between the two diseases, and its low expression was closely associated with innate immune activation, immunometabolic dysregulation, and enrichment of chronic inflammatory-related pathways. Further analyses revealed that both PTLDS and RA exhibited immune imbalance characterized by increased neutrophil infiltration and reduced adaptive immune cell populations, while showing varying degrees of immune-inflammatory and metabolic reprogramming in ZNF83-associated pathways. These findings suggest that PTLDS and RA may share partial molecular mechanisms characterized by innate immune dysregulation and persistent inflammatory responses, with ZNF83 potentially serving as an important node linking immune-inflammatory responses and metabolic regulation. This study provides new insights into the potential mechanisms underlying persistent immune abnormalities in PTLDS and offers a reference for future functional validation of ZNF83 and related therapeutic investigations.

## Data Availability

Publicly available datasets were analyzed in this study. This data can be found here: GSE63085: https://www.ncbi.nlm.nih.gov/geo/query/acc.cgi?acc=GSE63085. GSE77929: https://www.ncbi.nlm.nih.gov/geo/query/acc.cgi?acc=GSE77929. GSE229449: https://www.ncbi.nlm.nih.gov/geo/query/acc.cgi?acc=GSE229449.
